# Antimicrobial Use during the SARS-CoV-2 Pandemic in a Greek Tertiary University Hospital

**DOI:** 10.3390/microorganisms12030623

**Published:** 2024-03-20

**Authors:** Dimitrios Biros, Sempastian Filippas-Ntekouan, Diamantina Limperatou, Angelos Liontos, Rafail Matzaras, Konstantina-Helen Tsarapatsani, Nikolaos-Gavriel Kolios, Christiana Pappa, Maria Nasiou, Eleni Pargana, Ilias Tsiakas, Valentini Samanidou, Lazaros Athanasiou, Revekka Konstantopoulou, Haralampos Milionis, Eirini Christaki

**Affiliations:** 1 1st Division of Internal Medicine & Infectious Diseases Unit, University General Hospital of Ioannina, Faculty of Medicine, University of Ioannina, 45500 Ioannina, Greece; dimitrisbiros@gmail.com (D.B.); sebastienfilippas@gmail.com (S.F.-N.); andalimperatou@gmail.com (D.L.); angelosliontos@gmail.com (A.L.); rafail.matz@gmail.com (R.M.); il.tsiakas@gmail.com (I.T.); valentinasmnsmn@gmail.com (V.S.); lazathanasiou@gmail.com (L.A.); revekkakon@gmail.com (R.K.); hmilioni@uoi.gr (H.M.); 2 School of Electrical and Computer Engineering, National Technical University of Athens, 15773 Athens, Greece; ktsarapatsani@gmail.com; 3 Faculty of Medicine, University of Ioannina, 45110 Ioannina, Greece; ngkolios99@gmail.com (N.-G.K.); christianapappa4@gmail.com (C.P.); md06549@uoi.gr (M.N.); md06655@uoi.gr (E.P.)

**Keywords:** antimicrobial stewardship, COVID-19, bacterial co-infection, antibiotic use

## Abstract

In cases of SARS-CoV-2 hospitalization, despite low bacterial co-infection rates, antimicrobial use may be disproportionately high. Our aim was to quantify such usage in COVID-19 patients and identify factors linked to increased antibiotic use. We retrospectively studied patients with SARS-CoV-2 infection who were hospitalized at our institution during the pandemic. In the initial two waves of the pandemic, antimicrobial use was notably high (89% in the first wave and 92% in the second), but it decreased in subsequent waves. Elevated procalcitonin (>0.5 μg/mL) and C-reactive protein (>100 mg/L) levels were linked to antibiotic usage, while prior vaccination reduced antibiotic incidence. Antimicrobial use decreased in the pandemic, suggesting enhanced comprehension of SARS-CoV-2′s natural course. Additionally, it was correlated with heightened SARS-CoV-2 severity, elevated procalcitonin, and C-reactive protein levels.

## 1. Introduction

Since the first cases of COVID-19 pneumonia in Wuhan, China back in December 2019 and the declaration of a pandemic by the World Health Organization (WHO) on 11 March 2020, more than 771 million confirmed cases of SARS-CoV-2 infection and approximately 6,977,000 deaths have been recorded. Nearly four years later, as the pandemic wanes, our understanding of the virus has significantly broadened.

Bacterial co-infections frequently accompany viral pulmonary infections, such as influenza, playing a substantial role in increased morbidity and mortality [1,2]. However, co-infections were reportedly low [1,3] among COVID-19 patients with rates of primary bacterial co-infection of 1–4% [1,3]. The situation seems to be analogous concerning fungal infections [3]. Several studies published during the pandemic concur that despite a low incidence of bacterial or fungal co-infections, a substantial number of patients received empirical antibiotic prescriptions [1,2,3,4]. The pandemic notably fueled the extensive use of antibiotics. A meta-analysis demonstrated that despite a low bacterial infection rate, antibiotics were prescribed to over 70% of patients. The majority of these prescriptions featured broad-spectrum agents such as fluoroquinolones and third-generation cephalosporins, underscoring an overall inappropriate escalation in antibiotic usage [1,3,4]. Certainly, a pandemic-era meta-analysis revealed bacterial co-infection in 3.5% of patients and secondary bacterial infection in 14.3%. Notably, co-infections were more prevalent in critically ill patients. The discordance between co-infection rates and the high prevalence of prescribed antibiotics was the most pronounced during the initial wave of the pandemic [4].

Antimicrobial therapy was primarily guided by inflammatory markers like CRP, PCT, and WBC, as these markers were more frequently observed in patients with primary co-infections compared to those without [1]. Ferritin and LDH levels were elevated in individuals with community-acquired infections compared to those without [5]. Positive blood cultures were observed in a limited number of cases [5]. The decision to initiate antimicrobial treatment was influenced by elevated leukocyte counts, heightened CRP levels, intensive care unit (ICU) admission, and the need for oxygen supplementation. These factors were considered in the context of potential concurrent bacterial co-infection [4]. Conversely, the general incidence of nosocomial bacterial co-infections (occurring 48 h after admission) was primarily linked to ICU admission, followed by an extended hospital stay and a higher mortality rate [1,2,5].

Indeed, the widespread use of antimicrobial therapy has raised concerns regarding the escalating rates of antimicrobial resistance (AMR). The extensive utilization of last-line antibiotics in certain settings, coupled with the ongoing influx of hospitalized COVID-19 patients, poses a significant risk of substantial AMR escalation [3]. Coordinated efforts, aligned with antimicrobial stewardship practices, should strive to restrict unnecessary antibiotic usage and reduce the duration of antimicrobial administration [6]. As mentioned earlier, excluding the potential presence of co-infection or secondary bacterial pneumonia in hospitalized patients could be challenging [7], especially during initial assessment; this may still fuel the widespread prescription of antimicrobials in COVID-19 patients, posing a notable obstacle to antimicrobial stewardship programs [8]. 

In our article, we examined certain risk factors, including inflammatory markers, linked to the initiation of empiric antimicrobial treatment in a cohort of hospitalized patients with COVID-19. Our objective is to underscore potential markers that could facilitate a more targeted approach to antimicrobial treatment, aligning with antimicrobial stewardship principles and aiming to mitigate the further emergence of antimicrobial resistance.

## 2. Materials and Methods

### 2.1. Data Collection

We enrolled COVID-19 hospitalized patients from the Infectious Diseases Unit at the University Hospital of Ioannina, Greece. Data, including laboratory, somatometric, and demographic information, were gathered upon admission. SARS-CoV-2 infection diagnosis was confirmed by reverse transcriptase-polymerase chain reaction (RT-PCR) testing of nasopharyngeal swab specimens in all patients. The study spanned from March 2020 to September 2022 and was conducted in accordance with the highest standards outlined in the European Guidelines for Good Clinical and Laboratory Practice and the Helsinki Declaration. Patient records were anonymized and entered into a digital database. The pandemic waves were categorized as follows^.^ first wave: March 2020–August 2020, second wave: September 2020–December 2020, third wave: January 2021–July 2021, fourth wave: August 2021–December 2021, and fifth wave: January 2022–September 2022. The total population was 1353, with patients from the initial two waves excluded from the risk factor analysis due to exceptionally high antibiotic usage. The final study population for risk factor analysis comprised 1107 patients (January 2021/September 2022). 

### 2.2. Statistical Analysis

We performed an analysis using IBM SPSS Statistics 26, employing Pearson’s chi-square for dichotomous variables and Mann–Whitney U tests for continuous variables as initial assessments. Additionally, multivariate binary logistic regression was conducted, and the results are displayed in the tables below. The presented odds ratios are adjusted for age and sex through logistic regression.

### 2.3. Outcomes

We gathered essential demographic data (including age, gender, height, weight, and calculated BMI), comorbidities, and vaccination statuses. The primary parameters under study included CRP > 100 mg/L (C-reactive protein), PCT > 0.5 pg/mL (procalcitonin), WBC count >11,000/μL (white blood cell), PFR < 200 (PO_2_/FiO_2_), and CT burden of disease >50% (CTBoD; percentage of affected pulmonary parenchyma on chest computed tomography). Additionally, we assessed prior vaccination status against SARS-CoV-2, immunosuppression of any etiology, and prior corticosteroid use. The study’s outcome was defined as the initiation of antibiotics within the first 72 h of hospitalization.

## 3. Results

### 3.1. Study Population Characteristics

The study population’s basic characteristics are presented in Table 1.

Given the limited number of patients in the initial two pandemic waves, the final population did not markedly differ from the overall population in terms of both numbers and basic characteristics. The mean age of the final population was approximately 65 years, with an average BMI of 28.8 kg/m^2^. Vaccination rates were comparable between the two groups, reflecting the absence of vaccines at the onset of the pandemic. The most prevalent comorbidity was arterial hypertension (AH) in almost half of the population in both groups. Dyslipidemia was the second most prevalent comorbidity in both groups (in almost 1/3 of the population), while almost a quarter of the population suffered from diabetes mellitus (DM) as well. Obesity was also common, found in more than a quarter of the population, while smoking was less common, found in just over 10% of the population. The study groups did not differ in terms of the aforementioned comorbidities or other comorbidities, such as coronary artery disease (CAD), thyroid disease, pulmonary disease, autoimmune disease, chronic kidney disease (CKD), and cancer. 

### 3.2. Antibiotic Use during the Waves of the Pandemic

**Figure 1:**
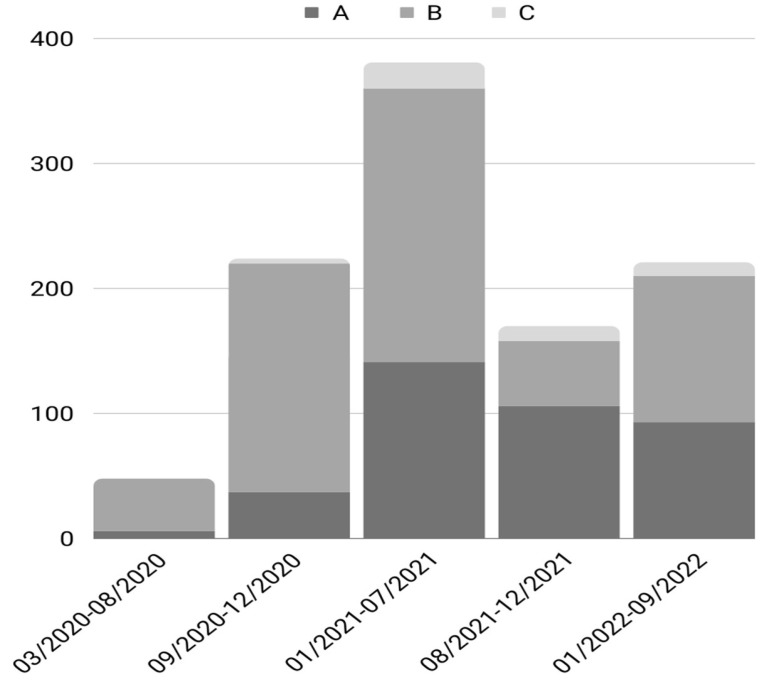
Bar chart of antibiotics administration during the waves of the pandemic. A: Outpatient antibiotics administration (prior to hospitalization), B: Antibiotic administration within 72 h of hospitalization, and C: Antibiotic administration after 72 h of hospitalization.

The frequency of antibiotics administration, both at home and during hospitalization, for each pandemic wave is presented in Table 2 and Figure 1.

Antibiotic administration was recorded at three timepoints: before hospitalization (outpatient), within 72 h of hospitalization, and after 72 h. From Table 2 and Figure 1, it is evident that inpatient antibiotic administration was high during the first two waves, while gradually it decreased in the later waves, when antibiotics were more frequently administered in outpatients.

### 3.3. Risk Factors for Antibiotic Usage

The risk factor analysis results are presented in Table 3.

Increased levels of CRP and procalcitonin were associated with higher rates of antimicrobial treatment initiation (OR = 2.71, *p* < 0.001 and OR = 3.96, *p* < 0.001, respectively). Similarly, CTBoD and PFR were also associated with an increased rate of antibiotic administration (OR = 2.10, *p* < 0.001 and OR = 1.61, *p* = 0.001). On the other hand, prior vaccination decreased the need for antibiotics (OR = 0.56, *p* < 0.001), while corticosteroid use and immunosuppression were not significantly associated with antimicrobial initiation. WBC count did not increase the rate of antibiotic administration significantly (OR = 1.33, *p* = 0.063).

## 4. Discussion

In our study, we aimed to explore the factors influencing empiric antimicrobial use in a cohort of hospitalized patients during the COVID-19 pandemic. Notably, we observed elevated antimicrobial usage within the first 72 h of admission in the initial two waves of the pandemic (89% in the first wave and 92% in the second wave). However, this trend decreased in subsequent waves (53% in the third wave, 18% in the fourth wave, and 29% in the fifth wave). On the other hand, outpatient antimicrobial use before hospital admission was higher during the subsequent pandemic waves and declined only in the fifth wave (13% in the first wave, 19% in the second wave, 34% in the third wave, 37% in the fourth wave, and 23% in the fifth wave). The increased outpatient antimicrobial use in the third and fourth waves probably reflected the increased number of patients seeking primary health care advice before their hospitalization. During the first two waves, due to public lockdowns, patients only visited acute care facilities for medical advice and were then either discharged or admitted. In the subsequent waves, patients consulted their primary health care providers and antimicrobials were often prescribed. However, inpatient antibiotic use was reduced after the first two waves, probably reflecting increased knowledge of the low co-infection rates and the increased experience of the medical staff in treating patients with COVID-19 infections. Furthermore, national guidelines did not recommend the routine use of antibiotics in patients with SARS-CoV-2 infection unless there were strong suspicions of bacterial co-infection. Regarding inpatients, the antimicrobial initiation rate after the third day of hospitalization did not show a significant difference between the waves of the pandemic.

These findings align with those reported by various investigators during the course of the COVID-19 pandemic. In a study of 3221 hospitalized patients with COVID-19 in Pakistan, [4], the vast majority (89.7%) of patients received antibiotics during their hospital stay, a percentage that remained high for all waves of the pandemic, despite the fact that only a small minority had a documented bacterial co-infection (1.1%) or a secondary bacterial infection (3.14%). Furthermore, antibiotic use within the first day of hospitalization remained high in all waves of the pandemic (in approximately 84% of the patients). However, the mean number of antibiotics used for every patient differed significantly between that after the second wave and that of the first two waves (2.03 ± 1.01 and 2.07 ± 0.87 antibiotics/patient in the first and second waves, respectively, compared with the fourth and fifth waves with an average of 1.35 ± 0.64 and 1.23 ± 0.81 antibiotics/patient, respectively). Duration of antibiotic use did not differ during the waves of the pandemic. In this study, the most commonly identified bacterial co-infections were *Staphylococcus aureus*, followed by *Streptococcus pneumoniae* and *Haemophilus influenzae*. Furthermore, the most common microorganisms identified in secondary bacterial infections were *Pseudomonas aeruginosa*, followed by *Streptococcus pneumoniae*, *Staphylococcus aureus*, *Escherichia coli*, and *Haemophilus influenzae*. The most commonly prescribed antibiotics were ceftriaxone, piperacillin + an enzyme inhibitor, azithromycin, and meropenem, followed by various other antibiotics in smaller percentages. In a study from the Philippines, [5] involving 1156 COVID-19 patients, within the initial 2 days of hospitalization, 55.1% received antibiotics, yet only 5.9% had documented community-acquired bacterial infection. Consistent with these findings, a review indicated that 72.1% of hospitalized COVID-19 patients received antibiotics [9]. After the second wave, patients received significantly fewer antimicrobials. Increased antimicrobial consumption was reported in another study, during the first COVID-19 wave [10]. Henig et al. [11] studied a cohort of 1836 hospitalized COVID-19 patients, in which there was a significant decrease in the proportion of patients receiving antibiotics from the first wave to the third wave (37.8% in the first wave compared to 30.5% in the third wave, *p* = 0.1). Additionally, the median duration of antibiotic treatment was notably shorter in the third wave than that in the first wave (a median of 6.5 days in the first wave compared to a median of 4 days in the third wave, *p* = 0.003). Importantly, this reduction in antibiotic use did not correlate with excess mortality during the waves of the pandemic. The most commonly used antimicrobials were second- and third-generation cephalosporins (52.2%), followed by agents with antipseudomonal activity (28%), vancomycin (16%), and aminoglycosides (11%). Furthermore, the most common reason for initiating antimicrobials was respiratory tract infection (62%), followed by urinary tract infection (18%) and sepsis (18%).The noteworthy decline in antibiotic usage can be attributed to an enhanced understanding of COVID-19 pathogenesis and mounting evidence indicating low co-infection rates with bacterial pathogens as the pandemic advanced. In another study from Greece that assessed bacterial infections in patients with COVID-19 [12], out of 177 participants in the third and fourth waves of the pandemic, 47 had at least one documented co-infection or superinfection. However, of these, only nine were classified as community-acquired infections. Only patients with late hospital-acquired infections had an increased mortality rate. However, details of antimicrobial usage were not provided in this study.

In an international point prevalence study [3], 62% of patients received antimicrobials during the first 48 h of admission, while their use was most commonly empirical (79.4%). Pneumonia was the leading indication of antimicrobial initiation during the first 48 h of admission (88% of indications). This percentage declined in patients receiving antimicrobials after 48 h of admission as only 45.3% of indications were listed as respiratory tract infection in this group of patients in medical wards, while the rate of other nosocomial infections increased. In intensive care units, pneumonia remained the main indication for empirical antimicrobial treatment (concerning 75.5% of prescriptions). Clinical presentation was the main reason for initiating antimicrobial treatment according to the attending physicians, followed by laboratory findings and radiological findings. The majority of the antibiotics prescribed were second- to fourth-generation cephalosporins, antipseudomonal b-lactams combined with a beta lactamase inhibitor, carbapenems, quinolones, macrolides, and vancomycin. In line with our results, a study encompassing 7209 patients with PCR-confirmed COVID-19 across three New York hospitals reported comparable findings [13], with antimicrobials being initiated in 70.1% of the patients. The predominant antimicrobials prescribed were third-generation cephalosporins (45.9%), closely followed by azithromycin (43.4%). A substantial number of patients (16.4%) received over three classes of antibiotics while a quarter of the patients received an antipseudomonal beta lactam. During the initial wave of the pandemic, 82.4% of patients received antimicrobials, compared to 52% in subsequent periods. Blood cultures were drawn in almost half of the patients, with sputum cultures collected in 6.1% of cases. Positive results were observed in 3.3% of blood cultures and 21% of sputum cultures. *Staphylococcus aureus* was consistently identified as the most common pathogen in both the blood and sputum cultures. *Escherichia coli* was the second most common organism identified in blood cultures, while as expected, *Streptococcus pneumoniae* was the second most common identified organism in sputum cultures, followed by *Pseudomonas aeruginosa*. Viral co-infections were exceedingly rare. In a systematic review and meta analysis [14], bacterial co-infection was reported in 7% of hospitalized COVID-19 patients. *Mycoplasma pneumoniae* was the most frequent organism identified in these patients (42%), followed by *Pseudomonas aeruginosa* and *Haemophilus influenzae*. Multiple other pathogens were identified in small percentages, such as *Klebsiella pneumoniae*, *Acinetobacter baumanii*, Chlamydia species, *Enterococcus faecium*, *Staphylococcus aureus*, and *Serratia marscecens*. Patients with an identified bacterial co-infection were more likely to die compared with patients with no bacterial co-infection (pooled OR 5.82, I^2^ 85.4%). In another systematic review and meta-analysis [15] of 75,956 confirmed COVID-19 patients, only 4.7% had a confirmed bacterial co-infection, while a random effects meta-analysis of all the combined studies estimated the prevalence of bacterial co-infection to be 5.62%, although with considerable heterogenicity. In the context of antimicrobial use, a random-effects meta-analysis across all the included studies estimated a prevalence of 61.16% for antibiotic prescriptions during the COVID-19 pandemic, marked by significant heterogeneity. Notably, North America exhibited the highest antimicrobial use, followed by Europe and Asia, also characterized by substantial heterogeneity.

In our cohort study, we aimed to discern the factors influencing the initiation of antimicrobials. Notably, WBC count did not significantly correlate with an increased incidence of antibiotic administration, compared with CRP and PCT levels. Similarly, high CTBoD and low PO_2_/FiO_2_ exhibited correlations with an increased rate of antibiotics, while vaccination was shown to be protective. Our findings align with similar results reported in other studies [4]. Following the second wave, there was a notable decrease in antimicrobial prescriptions for patients. Antimicrobials were more prevalent in individuals with higher WBC levels, increased CRP levels, heightened COVID-19 severity, and those requiring supplemental oxygen. Third-generation cephalosporins emerged as the most frequently prescribed antibiotic, followed by piperacillin/tazobactam, fluoroquinolones, macrolides, and carbapenems. In a prospective observational cohort study [1] of 309 hospitalized patients with COVID-19, 26.8% received antibiotics within the initial 48 h of hospitalization, with nearly half of them undergoing combination treatment. Those administered antimicrobials exhibited significantly higher levels of WBC, PMNs, CRP, and procalcitonin and were more likely to necessitate invasive ventilation. Documented bacterial co-infections, as evidenced by positive blood cultures or positive nasopharyngeal swabs, were extremely unlikely. However, in these cases, the patients exhibited higher CRP and procalcitonin levels. In another study of 1705 COVID-19 patients [16], 56.6% received empiric antimicrobial treatment, while only 3.5% had confirmed bacterial infection. Antibiotic usage was more prevalent among older patients, those with a lower BMI, or individuals displaying a lobar infiltrate.

Procalcitonin has served as a guide for both initiating and de-escalating antimicrobial treatment. Our findings indicate an association between higher procalcitonin levels and antimicrobial usage. In an observational study of 793 patients with COVID-19 [17], 28.2% were started on antibiotics. Among these, 14.7% had confirmed bacterial pneumonia, while 55.8% had suspected bacterial pneumonia. Initial procalcitonin levels were positively correlated with antibiotic initiation (OR = 1.23; 95% CI, 1.17–1.30; *p* < 0.001). In patients receiving antibiotics, median procalcitonin levels were 0.20 in those without bacterial pneumonia, 0.65 in those with possible pneumonia, and 0.88 in those with probable or proven bacterial pneumonia. These results correspond with an OR = 1.12 (95% CI, 1.03–1.22) for possible bacterial pneumonia versus no bacterial pneumonia and 1.22 (95% CI, 1.09–1.36) for probable or proven bacterial pneumonia versus no bacterial pneumonia for every 50% increase in PCT. The median antibiotic duration was 3 days in the group without bacterial pneumonia, 7 days in the group possibly having bacterial pneumonia, and 9 days in the group probably having or proven to have bacterial pneumonia. Interestingly, an initial procalcitonin level >0.25 ng/mL was correlated with an increased mean duration of antimicrobial treatment in both the group without bacterial pneumonia and in the group possibly having bacterial pneumonia. On the other hand, initial levels >0.25 ng/mL were not correlated with an increased mean duration of antibiotics in the group probably having or proven to have bacterial pneumonia. In line with the above, initial procalcitonin levels and percentage changes in daily measurements were independently associated with the duration of antibiotic exposure, while they were not associated with the number of classes of antibiotics prescribed. In another study, initial procalcitonin levels were elevated in patients with confirmed or suspected bacterial pneumonia. Specifically, procalcitonin levels were higher in patients with COVID-19 and bacterial pneumonia (median 0.58 ng/mL) compared to those without bacterial pneumonia (median 0.15 ng/mL) [18]. In patients without bacterial pneumonia, an elevated procalcitonin level was associated with an increased duration of antibiotic treatment compared to patients with low procalcitonin levels. The duration of antimicrobial treatment was 1 day in patients with low procalcitonin levels, while the duration was 4 days in patients with high procalcitonin levels, *p* < 0.01. Interestingly, in another study [19], in patients with COVID-19 and higher baseline procalcitonin and CRP levels, 32% developed a secondary bacterial infection during their hospital stay. Only a small minority of patients hospitalized in medical wards developed a secondary bacterial infection (6.3%), while 40% of the patients with COVID-19 hospitalized in medical wards received empirical antimicrobial treatment. On the other hand, regarding patients treated in intensive care units, approximately half developed a secondary bacterial infection (55.8%). In the same study, when a procalcitonin (PCT) cut-off value of 0.55 ng/mL was applied, it demonstrated a sensitivity of 91% and a specificity of 81% in identifying secondary bacterial infections; on the other hand, using a C-reactive protein (CRP) cut-off value of 172 mg/L resulted in a sensitivity of 81% and a specificity of 76%.

Corticosteroid use has been associated with statistically significant lower levels of CRP compared with no corticosteroid use in patients with COVID-19 [20]. Furthermore, a non-significant trend towards lower procalcitonin levels was observed in the same study in patients receiving corticosteroids compared with patients who did not receive either steroids or tocilizumab. However, this study examined prolonged courses of steroids in inpatients, thus limiting its impact on our study, as in our population, patients may have received only small courses of corticosteroids before hospitalization (and thus before PCT measurement). Furthermore, we have not identified similar studies in Greece studying the use of procalcitonin in patients with COVID-19. Therefore, while procalcitonin and CRP can complement clinical assessments for initiating or discontinuing antibiotic treatment, further evidence is essential to substantiate their role in distinguishing bacterial co-infection in hospitalized patients with COVID-19.

Our findings should be interpreted in the context of certain limitations. By design, this was a retrospective observational study, limiting its generalizability. Although we tried to study the most important factors associated with antimicrobial initiation, it is possible that other factors may have played a role.

## 5. Conclusions

Antimicrobial use, especially empiric prescriptions, exhibited a significant decline during the COVID-19 pandemic. This reduction likely reflects our evolving insights over the course of the pandemic and the accumulating amounts of scientific evidence indicating a relatively low incidence of bacterial co-infections in COVID-19 patients. Increased COVID-19 severity, as indicated by clinical parameters, laboratory findings, or chest imaging, was the primary factor associated with antimicrobial use. Our results can aid clinicians in deciding whether a patient presenting with COVID-19 infection is in need of antibacterial treatment. Furthermore, our results can inform the design of an antimicrobial stewardship program either at a local or national level. The implementation of antimicrobial stewardship programs holds promise in curbing antibiotic misuse and addressing the growing threat of infections caused by antimicrobial-resistant organisms.

## Figures and Tables

**Table 1:** Patient baseline characteristics for the total population and the final studied population for risk factor analysis; BMI—body mass index, AH—arterial hypertension, DM—diabetes melitus, CAD—coronary artery disease, CKD—chronic kidney disease.

	Total (N = 1353)		Final Population (N = 1107)	
	(n)	(%)	(n)	(%)
Demographics				
Gender (male/female)	748/575	56.6/43.4	630/477	56.9/43.1
Age (mean years)	65.2	-	64.8	-
BMI (mean kg/m^2^)	28.8	-	28.8	-
Vaccination	439	33.2	438	39.6
Comorbidities—risk factors				
AH	635	48.0	530	47.9
Dyslipidemia	466	35.2	396	35.8
DM	306	23.1	256	23.1
CAD	263	19.9	206	18.6
Thyroid disease	152	11.5	129	11.7
Pulmonary disease	96	7.3	83	7.5
Autoimmune disease	90	6.8	76	6.9
CKD	91	6.9	80	7.2
Cancer	113	8.5	96	8.7
Smoking	174	13.2	138	12.5
Obesity	344	26.0	298	26.9

**Table 2:** Frequency of antibiotic administration during the waves of the pandemic.

	Outpatient Antibiotics Administration	Antibiotics Administration within 72 h of Hospitalization	Antibiotics Administration after 72 h of Hospitalization
March 2020–August 2020 (N = 47)	6 (13%)	42 (89%)	0
September 2020–December 2020 (N = 199)	37 (19%)	183 (92%)	4 (2%)
January 2021–July 2021 (N = 412)	141 (34%)	219 (53%)	21 (5%)
August 2021–December 2021 (N = 290)	106 (37%)	52 (18%)	12 (4%)
January 2022–September 2022 (N = 405)	93 (23%)	117 (29%)	11 (3%)

**Table 3:** Binary logistic regression analysis results for antibiotic treatment initiation within 72 h of hospitalization. PCT—procalcitonin, CRP—C reactive protein, CTBoD—percentage of affected pulmonary parenchyma on computed tomography of the chest, WBC—white blood cell count, PFR—PO_2_/FiO_2_ ratio.

Risk Factor	OR	*p*-Value
PCT > 0.5 μg/L	3.96	<0.001
CRP > 100 mg/L	2.71	<0.001
CTBoD > 50%	2.10	<0.001
WBC > 11,000/μL	1.33	0.063
PFR < 200	1.61	0.001
Corticosteroid administration	0.97	0.881
Antibiotics administration at home	1.12	0.379
Immunosuppression	1.30	0.252
Vaccination	0.56	<0.001

## Data Availability

Data are not shared due to privacy issues.
